# Supplementary material for: Highly twisted supercoils for superelastic multi-functional fibres
Source: Nat Commun. 2019 Jan 25;10:426. doi: 10.1038/s41467-018-08016-w (PMC6347621; doi:10.1038/s41467-018-08016-w)
Supplement: Supplementary file 1 — Supplementary Information [file 41467_2018_8016_MOESM1_ESM.pdf]

Supplementary Information

Highly Twisted Supercoils for Superelastic Multi-Functional Fibers

Wonkyeong Son[1§], Sungwoo Chun[2§], Jae Myeong Lee[1], Yourack Lee[3], Jeongmin Park[3], Dongseok Suh[3], Duck Weon Lee[4], Hachul Jung[5], Young-jin Kim[5], Younghoon Kim[6], Soon Moon Jeong[1], Sang Kyu Lim[1] and Changsoon Choi[1]*

[1]Division of Smart Textile Convergence Research, DGIST, Daegu 42988, South Korea

[2]Department SKKU Advanced Institute of Nanotechnology (SAINT), Sungkyunkwan University, Suwon, Gyeonggi-do 16419, South Korea

[3]Department of Energy Science, Sungkyunkwan University, Suwon, Gyeonggi-do 16419, South Korea

[4]Center for Self-powered Actuation, Department of Biomedical Engineering, Hanyang University, Seoul 04763, South Korea

[5]Medical Device Development Center, Osong Medical Innovation Foundation, Cheongju, Chunbuk 28160, South Korea

[6]Convergence Research Center for Solar Energy, DGIST, Daegu 42988, South Korea

[§] These authors equally contributed to this work.

*To whom correspondence should be addressed. E-mail:cschoi@dgist.ac.kr

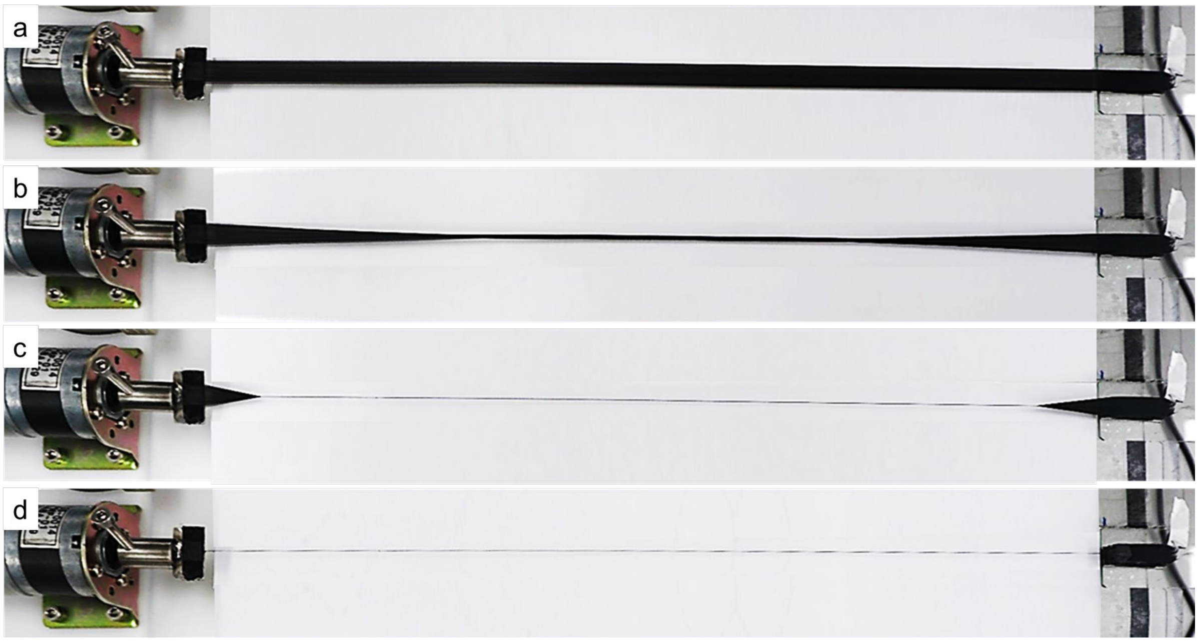

Supplementary Figure 1. Photographs of CNT sheets wrapping process on the stretched (ε_{fab} = 400%) spandex core fibre

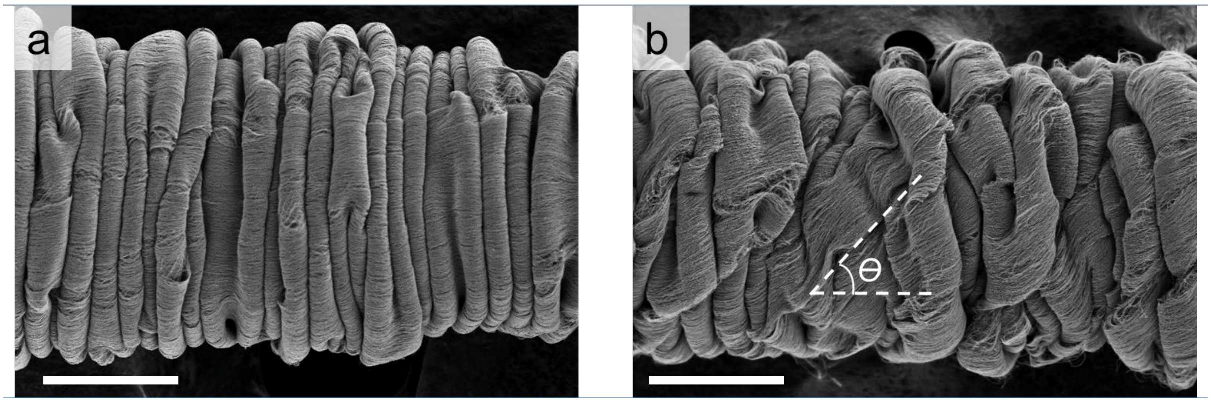

Supplementary Figure 2. SEM images of the spandex@CNT fibre (a) before, and (b) after twist insertion just before first-coil formation (scale bars = 100 μm).

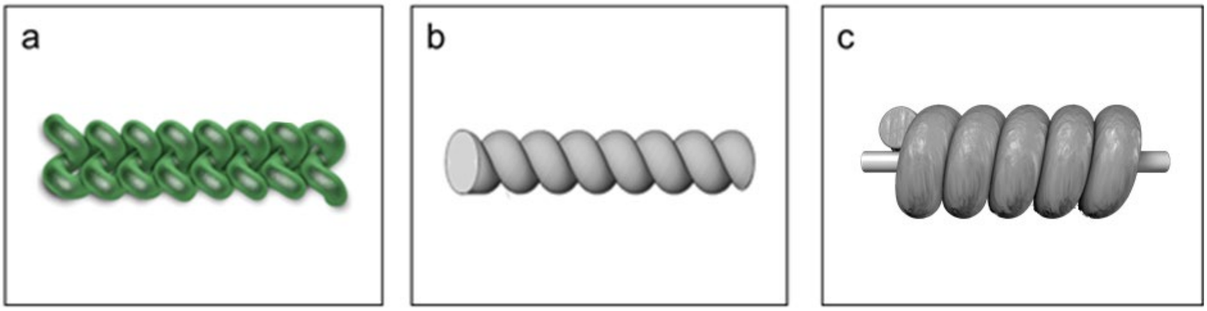

Supplementary Figure 3. Schematic illustrations of (a) supercoiled, (b) coiled, and (c) helical coiled fibres

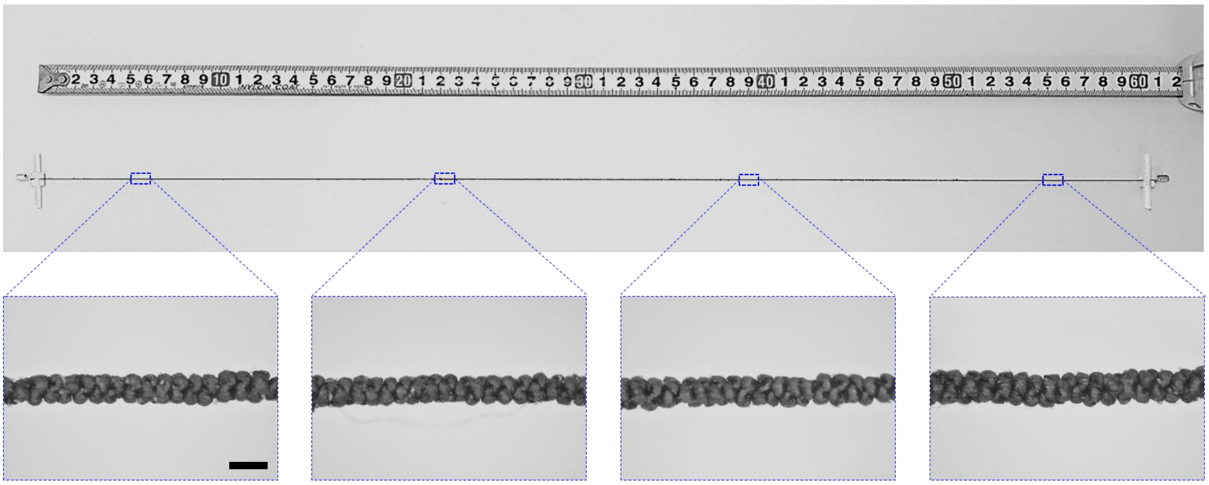

Supplementary Figure 4. Photograph (upper) and magnified optical images (lower) of four different spots on a 60 cm-long supercoil fibre (scale bar = 500 μm).

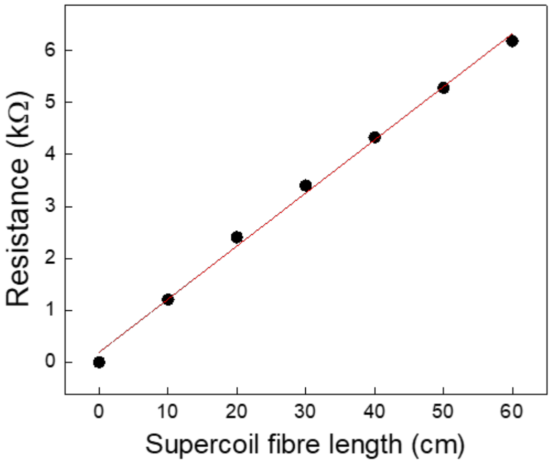

Supplementary Figure 5. Measured electrical resistance versus length of a 60 cm-long supercoil fibre.

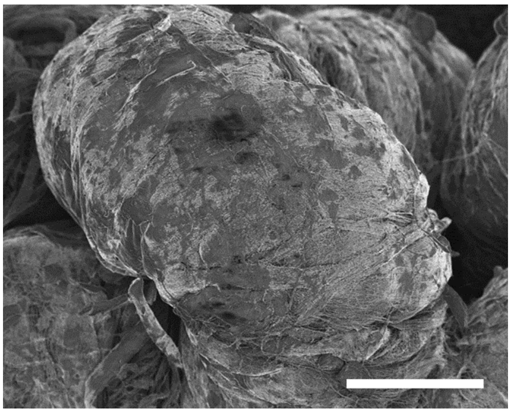

Supplementary Figure 6. SEM image of a SEBS-overcoated spandex@CNT supercoiled fibre (scale bars = 100 µm)

Supplementary Table 1. Quality factor (*Q*) comparison of presented supercoiled fibres with prior-art elastic yarn or fibre conductors

Structures and materials (Ref. No.)	Stretchability [$\Delta L/L_0$, %]	Resistance change [$\Delta R/R_0$, %]	Quality factor [$\Delta L \cdot R_0/\Delta R \cdot L_0$]
Spandex@ SEBS/CNT supercoiled fibres (present work)	1,000	4.19	238.8
(a) Buckled CNT@sandwich structured rubber fibre (1)	200	3.7	54
(b) Buckled CNT@SEBS fibre (2)	800		65.1
(c) CNT@nylon coiled fibre (3)	150	1373.7	0.109
(d) Buckled CNT@coiled rubber fibre (4)	400	221.7	1.8
(e) CNT@elastomer polymer fibre (5)	700	450	1.55

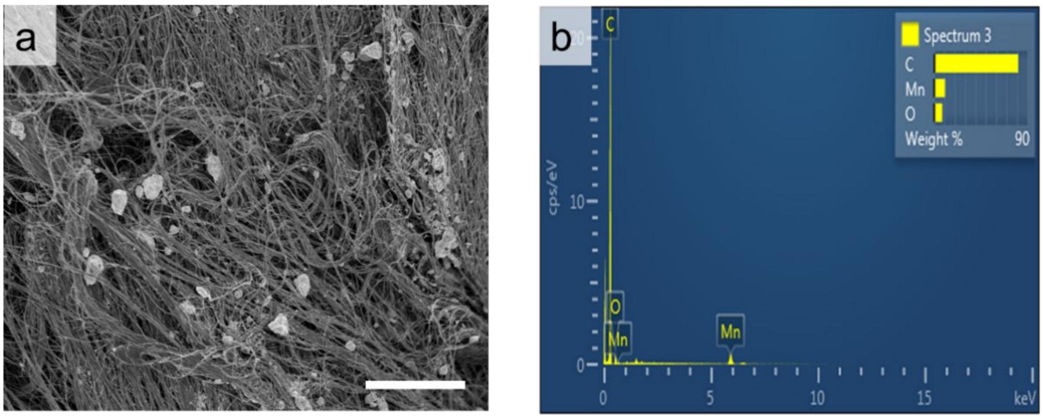

Supplementary Figure 7. (a) SEM image (scale bar = 10 µm), and (b) EDX elemental analysis of a MnO_2 coated spandex@CNT supercoiled fibre.

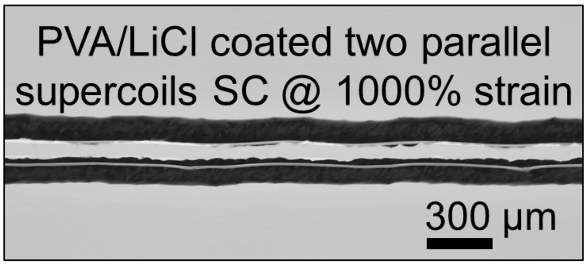

Supplementary Figure 8. Optical image of a supercoil based solid-state supercapacitor at 1000% tensile strain application, which comprises of two parallel, symmetric spandex@MnO_2/CNT supercoil fires and PVA/LiCl gel electrolyte coating (scale bar = 300μm).

Supplementary Table 2. Comparison of specific capacitance for the supercoil supercapacitor with prior-art yarn or fibre based elastic supercapacitors

**Electrode Materials (Ref. No.)**	**C_L [mF cm^{-1}]**	**C_A [mF cm^{-2}]**	**Stretchability [%]**
Spandex@MnO_2/CNT supercoiled fibres (this work)	21.7	92.1	1,000
MnO_2/CNT@coiled nylon fibres (3)	5.4	40.9	150
Buckled MnO_2/CNT@coiled rubber fibres (4)	4.8	22.8	400
Buckled MnO_2/CNT@sandwich structured rubber fibres (1)	2.4	11.9	200
PANI/CNT@elastomeric polymer fibres (5)	0.9	50.1	400
MnO_2/CNT@spandex, CNT@spandex asymmetric fibres (6)	0.26	27.9	100
CNT/graphene/PANI helical-coil fibres (7)	10.3	273.7	800
PEDOT-S:PSS helical-coil fibre (8)		93.1	400
SEBS@CNT/graphene/MnO_2 helical-coil fibres (9)		14	850

Supplementary References

1. Choi, C. *et al.* Twistable and stretchable sandwich structured fiber for wearable sensors and supercapacitors. *Nano Lett.* **16**, 7677-7684 (2016)

2. Wang, H. *et al.* Downsized sheath–core conducting fibers for weavable superelastic wires, biosensors, supercapacitors, and strain sensors. *Adv. Mater.* **28**, 4998-5007 (2016)

3. Choi, C. *et al.* Stretchable, weavable coiled carbon nanotube/MnO_2/polymer fiber solid-state supercapacitors. *Sci. Rep.* **5**, 9387 (2015)

4. Choi, C. *et al.* Microscopically buckled and macroscopically coiled fibers for ultra-stretchable supercapacitors. *Adv. Energy Mater.* **7**, 1602021 (2017)

5. Zhang, Z. *et al.* Superelastic supercapacitors with high performances during stretching. *Adv. Mater.* **27**, 356-362 (2015)

6. Xu, P. *et al.* Stretchable wire-shaped asymmetric supercapacitors based on pristine and MnO_2 coated carbon nanotube fibers. *ACS Nano* **9**, 6088-6096 (2015)

7. Lu, Z., Foroughi, J., Wang, C., Long, H. & Wallace, G. G. Superelastic hybrid CNT/graphene fibers for wearable energy storage. *Adv. Energy Mater.* **8,** 1702047 (2018)

8. Wang, z. *et al.* All-in-one fiber for stretchable fiber-shaped tandem supercapacitors. *Nano Energy* **45,** 210-219 (2018)

9. Wang, H. *et al.* Superelastic wire-shaped supercapacitor sustaining 850% tensile strain based on carbon nanotube@graphene fiber. *Nano Research* **11,** 2347-2356 (2018)
