# Supplementary material for: Highly twisted supercoils for superelastic multi-functional fibres
Source: Nat Commun. 2019 Jan 25;10:426. doi: 10.1038/s41467-018-08016-w (PMC6347621; doi:10.1038/s41467-018-08016-w)
Supplement: Supplementary file 3 — Description of Additional Supplementary Files [file 41467_2018_8016_MOESM3_ESM.pdf]

Description of Additional Supplementary Files

File Name: Supplementary Movie 1
Description: Stretchable sound transmission line made of supercoiled spandex@CNT fibre

File Name: Supplementary Movie 2
Description: Stretchable video transmission line made of supercoiled spandex@CNT fibre
